# Identification of novel immune checkpoints as targets for cancer immunotherapy

**DOI:** 10.1186/2051-1426-2-S3-P110

**Published:** 2014-11-06

**Authors:** Ofer Levy, Amir Toporic, Gady Cojocaru, Liat Dassa, Ilan Vaknin, Tal Fridman Kfir, Inbal Barbiro, Eyal Neria, Arthur Machlenkin, Spencer Liang, Shirley Sameach-Greenwald, Zurit Levin, Galit Rotman, John Hunter

**Affiliations:** 1 Compugen Ltd.,Israel; 2 Compugen Inc., San Francisco, CA, USA

## 

Members of the B7/CD28 family of immune checkpoints, such as CTLA4, PD1 and PDL-1, play critical roles in T cell regulation, and have emerged as promising drug targets for cancer immunotherapy. We hypothesize that additional novel members of the B7/CD28 family play a role as negative immune regulators, and thus may serve as targets for therapeutic mAbs. Utilizing Compugen's predictive discovery platform, we identified eleven novel proteins that may serve as potential immune checkpoint candidates. The therapeutic potential of four of these proteins, CGEN-15001T, CGEN-15022, CGEN-15049 and CGEN-15052, was confirmed following validation of their immunomodulatory properties and demonstration of their expression in various cancers. Here we present results for two of these novel immune checkpoint candidates, CGEN-15049 and CGEN-15052.

A recombinant protein consisting of the extracellular domain of CGEN-15049 fused to an IgG Fc domain was shown to inhibit T cell activation and enhance iTregs differentiation. Moreover, ectopically expressed CGEN-15049 inhibits activation of melanoma-specific CTLs and dampens function of NK cells, supporting a role for this protein in tumor immune modulation. IHC studies indicate that CGEN-15049 is expressed in the tumor cells and in the tumor infiltrating immune cells of multiple epithelial cancers.

CGEN-15052 has demonstrated robust inhibition of T cell activation in several experimental settings, both as an ectopically expressed membrane protein and as an Fc fusion protein. IHC studies indicate that CGEN-15052 is expressed in multiple epithelial cancers, with particularly high expression in lung cancer samples.

Taken together, the ability of both of these proteins to modulate immune response in several types of cells that play key roles in cancer immune evasion, and their tumor expression profile, indicate that CGEN-15049 and CGEN-15052 are potential immune checkpoints that may serve as targets for cancer immunotherapy.

